# Commentary: Presence of kratom in opioid overdose deaths: findings from coroner postmortem toxicological report

**DOI:** 10.3389/fpsyt.2024.1411964

**Published:** 2024-05-23

**Authors:** Oliver Grundmann, Kirsten E. Smith, Walter C. Prozialeck, Charles A. Veltri, Edward W. Boyer

**Affiliations:** ^1^ Department of Medicinal Chemistry, College of Pharmacy, University of Florida, Gainesville, FL, United States; ^2^ Department of Psychiatry and Behavioral Sciences, School of Medicine, Johns Hopkins University, Baltimore, MD, United States; ^3^ Department of Pharmacology, Midwestern University, Downers Grove, IL, United States; ^4^ Department of Pharmaceutical Science, College of Pharmacy, Midwestern University, Glendale, AZ, United States; ^5^ Department of Emergency Medicine, The Ohio State College of Medicine, Columbus, OH, United States

**Keywords:** kratom, mitragynine, toxicology, safety, public health

## 1 Introduction

A recent publication in *Frontiers in Psychiatry, Addictive Disorders* described the forensic analysis of four polydrug fatalities involving kratom in a California county (1). Kratom products derived from the botanical kratom (*Mitragyna speciosa* Korth.) have gained attention in the United States due to such reports of adverse effects and fatalities associated with their use. The tree from which various products are prepared is native to Southeast Asia where the leaves have been used for centuries to self-treat a variety of ailments (2). Kratom products in the US are estimated to be used by as many as 15 million adults depending on the source of the estimate (3, 4). While unregulated at the federal level in the US, kratom is regulated and considered an illicit novel psychoactive substance in several European and Southeast Asian nations (5, 6). Some of the pharmacological actions of kratom alkaloids, including kratom’s major alkaloid, mitragynine, are mediated through weak agonist activity at opioid receptors, although alpha-adrenergic and serotonergic receptors are also involved (7).

Torrico and colleagues described the forensic analysis of four kratom-related fatal polydrug exposures in a California county (1). Their report, while correct in mentioning some of the context surrounding kratom consumption, incorrectly reported a blood concentration as a lethal dose, neither of which have been established, and lacked details critical to appropriately interpreting the causal contribution of kratom to toxicity or a fatal outcome. Here, we address some ambiguities in that report.

## 2 Context of kratom use

Complexities of kratom use confound interpretation of use-related risk or adverse events. One example is that kratom persists as a harm-reduction method to reduce opioid and other substance use (8, 9). Previously, some consumers with opioid use disorder (OUD) history reported that kratom is not preferred over illicit opioids and do not report using kratom to achieve a euphoric “high” (10, 11). More recently, among adults who use kratom regularly and prefer it to other substances, most do not use to become intoxicated or “high”, even among people with OUD history (12). Still, within the broader kratom-using population, the subset of consumers with OUD history remain at risk for relapse. As such, the risk-benefit ratio of kratom is complicated. Kratom is also used nonmedically to attenuate withdrawal symptoms from opioids, stimulants and alcohol (13, 14). One survey found that a majority (94.8%) of kratom consumers had abstained from other substance use for at least 6 months, highlighting kratom’s role in nonmedically managing addiction (11, 12, 15, 16).

The US Food and Drug Administration (FDA), which classifies kratom products as new dietary ingredients that require proof of safety before they can enter the market, has issued an import alert that designates kratom-containing products as adulterated and subject to seizure (17). Indeed, some kratom products sold in the US have been found to be adulterated with excessive amounts of kratom alkaloids, in particular the more potent mitragynine metabolite 7-hydroxymitragynine which is not present in fresh kratom leaves (18, 19).

## 3 Fatalities involving kratom use

Kratom contains a complex mixture of phytochemicals that produce multiple pharmacological effects. As the most abundant alkaloid in kratom, mitragynine has been used by forensics laboratories as the indicator of exposure (20, 21). Torrico et al. incorrectly state that the lethal dose of mitragynine was 0.398 mg/L based on three cases (1). The mitragynine concentration quoted by Torrico et al. is not a *dose* but rather a *blood concentration* identified in adults who had mitragynine in their blood. These authors are not alone in reaching the erroneous conclusion that this would be a lethal concentration (20). It is noteworthy that no causative lethal blood concentration, nor a lethal *dose* for kratom, mitragynine, or other alkaloids, has been established in humans or animals (22). The concentration identified by Torrico et al. is even lower than that found in another study of 6,860 post-mortem blood samples that tested positive for mitragynine (21). This study concluded that mitragynine blood concentrations >1,000 ng/mL are more often associated with severe adverse events, including death. Of note, post-mortem 7-hydroxymitragynine levels are more likely than mitragynine to be a theoretically robust indicator of causality with respect to kratom-related toxicity and death given its relative potency (23). Unfortunately, 7-hydroxymitragynine is unstable in biological samples, thus complicating its interpretive value given variable post-mortem intervals before sample collection and analysis (24). Furthermore, most fatalities associated with kratom involve polydrug exposures that complicate assigning causation to mitragynine (8, 25). In addition, forensic cases involving only mitragynine may reflect incomplete toxicology testing. Thus, a death attributed to polydrug exposure may erroneously include kratom as a contributory but *not* causative agent, even if other substances are present in concentrations consistent with subtherapeutic, therapeutic, or overdose use.

## 4 Discussion

Increased awareness of kratom’s complex polypharmacy by the public, clinicians, and public health officials requires accurate information on the current state of scientific knowledge. A diversity of commercial kratom products exists, including highly concentrated formulations with many times the amount of mitragynine and minor alkaloids than exist in native leaf material. The sheer range of commercial products is paralleled by a diversity of recommended serving sizes. This complexity may heighten the risk of using compositions that contain the more potent metabolite 7-hydroxymitragynine (26). While federal regulation and oversight are lacking, the US kratom industry has been left to regulate itself, primarily at the state level (5).

For now, kratom-only fatalities, although rare, must be taken in the context of the specific commercial product used, previous medical and drug use history, and the limitations of testing. The Torico et al. article follows several case study reports that did not document what kratom products were consumed and misinterpreted the mitragynine concentration as being a “dose.” Usual doses or servings of kratom leaf products range from 1-10 g containing between 20 and 200 mg mitragynine while concentrated kratom extracts can contain many times that amount of mitragynine. Although sufficient clinical data are lacking, kratom could be associated with adverse effects if taken in high amounts of leaf or concentrated extract, and at more frequent daily dosing intervals. Documenting clinically unremarkable use and adverse events associated with kratom remains critical. Federal oversight is needed to establish safe dosing recommendations across populations, and to apply a regulatory framework that ensures consumers have access to safe and properly labeled kratom products.

## Author contributions

OG: Writing – original draft, Writing – review & editing. KES: Writing – review & editing. WCP: Writing – review & editing. CAV: Writing – review & editing. EWB: Writing – review & editing.
